# Synchronization enhancement of indirectly coupled oscillators via periodic modulation in an optomechanical system

**DOI:** 10.1038/s41598-017-16115-9

**Published:** 2017-11-20

**Authors:** Lei Du, Chu-Hui Fan, Han-Xiao Zhang, Jin-Hui Wu

**Affiliations:** Center for Quantum Sciences and School of Physics, Northeast Normal University, Changchun, 130117 China

## Abstract

We study the synchronization behaviors of two indirectly coupled mechanical oscillators of different frequencies in a doublecavity optomechanical system. It is found that quantum synchronization is roughly vanishing though classical synchronization seems rather good when each cavity mode is driven by an external field in the absence of temporal modulations. By periodically modulating cavity detunings or driving amplitudes, however, it is possible to observe greatly enhanced quantum synchronization accompanied with nearly perfect classical synchronization. The level of quantum synchronization observed here is, in particular, much higher than that for two directly coupled mechanical oscillators. Note also that the modulation on cavity detunings is more appealing than that on driving amplitudes when the robustness of quantum synchronization is examined against the bath’s mean temperature or the oscillators’ frequency difference.

## Introduction

As one of the most attractive phenomena in physics and even the whole natural science, spontaneous synchronization of coupled oscillators has been explored with intense interests recently in various fields like nonlinear dynamics^1–6^, cavity optomechanics^7–11^, quantum information processing (QIP)^12,13^, Bose-Einstein Condensates^14^, atomic ensembles^15–17^ and so on. The synchronization phenomenon was discovered earliest in a classical clock pendulum system by Huygens in the 17*th* ury^18^ and has been successfully extended to the quantum regime now^19^, *e*.*g*., for realizing the synchronous manipulation of quantum information and quantum states. In particular, Yamada *et al*. proposed to use the Lyapunov index as a qualitative criterion in order to determine whether the classical synchronization is reached for coupled oscillators^20^. Subsequently, Mari *et al*. put forward an effective synchronization measure for continuous variable (CV) quantum systems^19^ with two directly coupled microscopic oscillators taken as a good example. Investigations on quantum synchronization in optomechanical systems soon achieved great success with relevant experiments done to verify the theoretical predictions^21–23^, which laid a favorable foundation for the further studies and applications.

According to the existing studies, synchronization behaviors between mechanical oscillators usually occur in two ways: (i) they exchange energy directly owing to an effective coupling so that their oscillations tend to be accordant after a long enough time^8,19^; (ii) they are restricted to evolve towards a generalized synchronization, *e*.*g*., by the Lyapunov control of external fields in the absence of a direct coupling^9,24^. But a mechanical oscillator may also be synchronized to a reference drive^25,26^, thereby allowing the synchronization of uncoupled mechanical oscillators in the presence of identical driving fields. Generally speaking, optomechanical systems with directly coupled oscillators have a stronger maneuverability in achieving quantum synchronization than those with indirectly coupled oscillators. That is, indirectly coupled oscillators typically exhibits more poor synchronization behaviors and involves more complicated control strategies than directly coupled oscillators. On the other hand, we note that proper time-periodic modulations can open new possibilities for achieving optimal quantum control strategies and has been used to enhance various quantum effects like squeezing and entanglement in optomechanical systems^27,28^. Then one essential question arises: may time-periodic modulations also help to enhance quantum synchronization of indirectly coupled oscillators? The main aim of this work is thus to seek a *positive* answer with the quantum synchronization measure approaching perfect (→1.0), far beyond that for directly coupled oscillators (~0.3)^19^.

Here we study the dynamic evolution of two mechanical oscillators interacting with different cavity modes via the radiation pressure in a double-cavity optomechanical system. The two cavities are coupled by an optical fiber through the inside mirrors and driven by two optical fields through the outside mirrors. It is shown that the two oscillators exhibit quite poor synchronization behaviors with the quantum part being negligible though the classical part being passable when the double-cavity optomechanical system suffers no temporal modulation. Exerting periodic modulations on detunings of both cavity modes or on amplitudes of both driving fields, we find that rather satisfactory synchronization behaviors can be observed with the quantum part being greatly enhanced and the classical part approaching perfect. To be more specific, the optimal quantum synchronization can be ~0.92 (~0.74) in the case of double cavity-detuning (driving-amplitude) modulation when the oscillators’ frequency difference is not too large at a low enough bath’s mean temperature. The advantage of cavity-mode modulation over driving-field modulation is further confirmed by an examination on the robustness of quantum synchronization against the bath’s mean temperature and the oscillators’ frequency difference.

## Model and Methods

**Figure 1:**
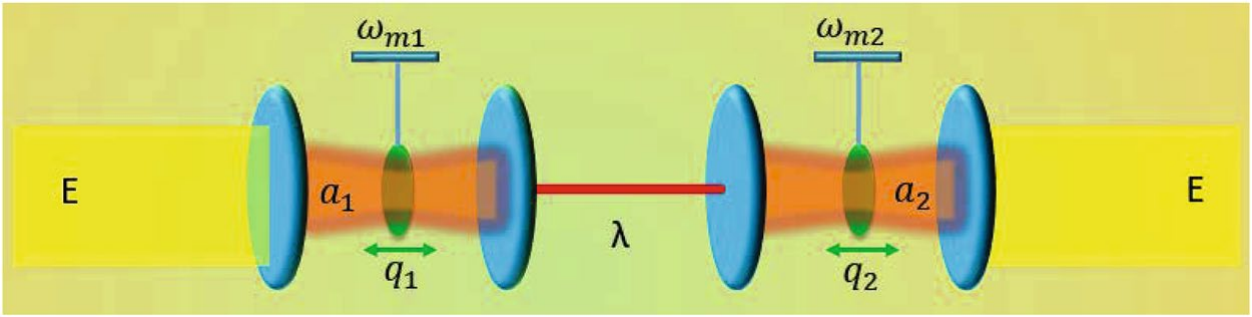
Schematic illustration of an optomechanical system with two mechanical oscillators of frequencies ${\omega }_{m\mathrm{1,}m2}$ contained in different optical cavities. Each cavity mode ${a}_{\mathrm{1,2}}$ may be excited by a driving field of amplitude *E* and result in a deviation *q*_1,2_ of one oscillator from its equilibrium position due to the radiation pressure. The two cavity modes are further coupled through an optical fiber described by the constant *λ*. One may adopt the piezoelectric effect to modulate the mode detunings or the acousto-optical effect to modulate the driving amplitudes so that the oscillators’ synchronization behaviors can be enhanced.

The optomechanical system under consideration is illustrated in Fig. 1. Two Fabry-Pérot cavities are coupled by an optical fiber between the inside mirrors and driven by two fields through the outside mirrors. Each cavity contains a tiny mechanical oscillator interacting with a corresponding cavity mode via the radiation pressure. A time-periodic modulation may be applied upon both external driving fields^27,29^ via the acousto-optical effect or both internal cavity modes via the piezo-electric effect^30^. Then it is straightforward to write down the total Hamiltonian after a frame rotating

1 $$\begin{array}{c}H=\sum _{j\mathrm{=1,2}}\{-{{\rm{\Delta }}}_{j}[1+{\eta }_{C}\,\cos ({{\rm{\Omega }}}_{C}t)]{a}_{j}^{\dagger }{a}_{j}+\frac{{{\rm{\omega }}}_{mj}}{2}({p}_{j}^{2}+{q}_{j}^{2})-g{a}_{j}^{\dagger }{a}_{j}{q}_{j}\\ \,\,\,\,\,\,+iE[1+{\eta }_{D}\,\cos ({{\rm{\Omega }}}_{D}t)]({a}_{j}^{\dagger }-{a}_{j})\}+{\lambda }({a}_{1}^{\dagger }{a}_{2}+{a}_{2}^{\dagger }{a}_{1})\end{array}$$

where $\hslash =1$ has been set for convenience. We have also assumed that (i) the two driving fields have the same frequency *ω* and the same amplitude *E* (ii) the two driving fields (cavity modes) are modulated in the same way with a common frequency Ω_*D*_ (Ω_*C*_) and amplitude ${\eta }_{D}$ (${\eta }_{C}$). In addition, ${{\rm{\Delta }}}_{j}={\rm{\omega }}-{{\rm{\omega }}}_{cj}$ is the detuning of the *j* th cavity mode with ${\omega }_{cj}$ being the mode frequency; ${\omega }_{mj}$ is the frequency of the *j* th mechanical oscillator; ${a}_{j}^{\dagger }$ (*a*_*j*_) is the creation (annihilation) operator of cavity mode ${\omega }_{cj}$, satisfying the commutation relation $[{a}_{j},{a}_{{j}^{\text{'}}}^{\dagger }]={\delta }_{j{j}^{\text{'}}}$; *q*_*j*_ (*p*_*j*_) is the dimensionless position (momentum) operator of mechanical oscillator ${\omega }_{mj}$, satisfying the commutation relation $[{q}_{j},{p}_{{j}^{\text{'}}}]=i{\delta }_{j{j}^{\text{'}}}$; *g* is the optomechanical coupling constant due to the radiation pressure and assumed to be equal in both cavities for simplicity; *λ* is the coupling constant of cavity modes through an optical fiber.

Using the above Hamiltonian and considering relevant dissipation processes, we can further attain the following quantum Langevin equations^27,31,32^

2 $$\begin{array}{rcl}{\dot{q}}_{j} & = & {\omega }_{mj}{p}_{j}\\ {\dot{p}}_{j} & = & -{\omega }_{mj}{q}_{j}-{\gamma }_{m}{p}_{j}+g{a}_{j}^{\dagger }{a}_{j}+{\xi }_{j}\\ {\dot{a}}_{j} & = & -\{\kappa -i{{\rm{\Delta }}}_{j}[1+{{\rm{\eta }}}_{C}{\cos ({\rm{\Omega }}}_{C}t)]\}{a}_{j}+ig{a}_{j}{q}_{j}\\  &  & +E[1+{{\rm{\eta }}}_{D}{\cos ({\rm{\Omega }}}_{D}t)]-i\lambda {a}_{3-j}+\sqrt{2\kappa }{a}_{j}^{in}\end{array}$$

with *k* being the common decay rate of both cavity modes while *γ*_*m*_ being the common damping rate of both mechanical oscillators. Moreover, ${a}_{j}^{in}$ describes the input noise operator of one cavity mode, exhibiting a zero mean value and satisfying the correlation relation $\langle {a}_{j}^{in\dagger }(t){a}_{{j}^{\text{'}}}^{in}({t}^{\text{'}})+{a}_{{j}^{\text{'}}}^{in}({t}^{\text{'}}){a}_{j}^{in\dagger }(t)\rangle ={\delta }_{j{j}^{\text{'}}}\delta (t-{t}^{\text{'}})$ ^33,34^; ${\xi }_{j}$ describes the stochastic noise operator of one mechanical oscillator, exhibiting a zero mean value and satisfying the correlation relation $\frac{1}{2}\langle {\xi }_{j}(t){\xi }_{{j}^{\text{'}}}({t}^{\text{'}})+{\xi }_{{j}^{\text{'}}}({t}^{\text{'}}){\xi }_{j}(t)\rangle ={\gamma }_{m}\mathrm{(2}{n}_{b}+\mathrm{1)}{\delta }_{j{j}^{\text{'}}}\delta (t-{t}^{\text{'}})$ under the Markovian approximation. Here ${n}_{b}={[exp(\hslash {\omega }_{m1}/{k}_{b}T)-1]}^{-1}\underline{ \sim }{[exp(\hslash {\omega }_{m2}/{k}_{b}T)-1]}^{-1}$ is the mean phonon number determined by the mechanical bath’s mean temperature *T* ^35–37^.

To solve Eq. (2), we adopt a mean-field approximation^8,9,27,28^ to express relevant operators as sums of the (large) mean values and the (small) fluctuation terms, *i*.*e*., ${o}_{j}={O}_{j}+\delta {o}_{j}$ with ${o}_{j}\in ({q}_{j},{p}_{j},{a}_{j})$. In this way, the quantum Langevin equations can be divided into a set of classical nonlinear differential equations

3 $$\begin{array}{rcl}{\dot{Q}}_{j} & = & {\omega }_{mj}{P}_{j}\\ {\dot{P}}_{j} & = & -{\omega }_{mj}{Q}_{j}-{\gamma }_{m}{P}_{j}+g|{A}_{j}{|}^{2}\\ {\dot{A}}_{j} & = & -\{\kappa -i{{\rm{\Delta }}}_{j}[1+{\eta }_{C}{\cos ({\rm{\Omega }}}_{C}t)]\}{A}_{j}+ig{A}_{j}{Q}_{j}\\  &  & +E\mathrm{[1}+{\eta }_{D}{\cos ({\rm{\Omega }}}_{D}t)]-i\lambda {A}_{3-j}\end{array}$$

for the mean values *O*_*j*_ and a set of quantum linear differential equations

4 $$\begin{array}{rcl}\dot{\delta }{q}_{j} & = & {\omega }_{mj}\delta {p}_{j}\\ \dot{\delta }{p}_{j} & = & -{\omega }_{mj}\delta {q}_{j}-{\gamma }_{m}\delta {p}_{j}+g({A}_{j}\delta {a}_{j}^{\dagger }+{A}_{j}^{\ast }\delta {a}_{j})+{\xi }_{j}\\ \dot{\delta }{a}_{j} & = & -\{\kappa -i{{\rm{\Delta }}}_{j}[1+{\eta }_{C}\,\cos ({{\rm{\Omega }}}_{C}t)]\}\delta {a}_{j}+ig({A}_{j}\delta {q}_{j}+{Q}_{j}\delta {a}_{j})\\  &  & -i\lambda \delta {a}_{3-j}+\sqrt{2\kappa }{a}_{j}^{in}\end{array}$$

for the fluctuation terms $\delta {o}_{j}$. In Eq. (4), we have neglected the second-order smaller terms including $\delta {a}_{j}^{\dagger }\delta {a}_{j}$ and $\delta {a}_{j}\delta {q}_{j}$. Further introducing $\delta {x}_{j}=(\delta {a}_{j}^{\dagger }+\delta {a}_{j}/\sqrt{2})$ and $\delta {y}_{j}=i(\delta {a}_{j}^{\dagger }-\delta {a}_{j})/\sqrt{2}$ as well as ${x}_{j}^{in}=({a}_{j}^{in\dagger }+{a}_{j}^{in})/\sqrt{2}$ and ${y}_{j}^{in}=i({a}_{j}^{in\dagger }-{a}_{j}^{in})/\sqrt{2}$, we can recast Eq. (4) into

5 $$\dot{u}=Mu+n$$

in terms of a 1 × 8 variable column vector $u=(\delta {q}_{1},\delta {p}_{1},\delta {x}_{1},\delta {y}_{1},\delta {q}_{2},\delta {p}_{2},\delta {x}_{2},\delta {y}_{2}{)}^{T}$, a 1 × 8 noise column vector $n={(\mathrm{0,}{\xi }_{1},\sqrt{2\kappa }{x}_{1}^{in},\sqrt{2\kappa }{y}_{1}^{in},\mathrm{0,}{\xi }_{2},\sqrt{2\kappa }{x}_{2}^{in},\sqrt{2\kappa }{y}_{2}^{in})}^{T}$, and a 8 × 8 coefficient matrix *M* given as follows

6 $${\bf{M}}=(\begin{array}{cc}{M}_{1} & {M}_{0}\\ {M}_{0} & {M}_{2}\end{array})$$

with

7 $${{\bf{M}}}_{{\bf{0}}}=(\begin{array}{cccc}0 & 0 & 0 & 0\\ 0 & 0 & 0 & 0\\ 0 & 0 & 0 & \lambda \\ 0 & 0 & -\lambda  & 0\end{array})$$

and

8 $${{\bf{M}}}_{1{\boldsymbol{,}}2}=(\begin{array}{cccc}0 & {\omega }_{m\mathrm{1,2}} & 0 & 0\\ -{\omega }_{m\mathrm{1,2}} & -{\gamma }_{m} & \sqrt{2}gRe({A}_{\mathrm{1,2}}) & \sqrt{2}gIm({A}_{\mathrm{1,2}})\\ -\sqrt{2}gIm({A}_{\mathrm{1,2}}) & 0 & -\kappa  & -{F}_{\mathrm{1,2}}\\ \sqrt{2}gRe({A}_{\mathrm{1,2}}) & 0 & {F}_{\mathrm{1,2}} & -\kappa \end{array})$$

where ${F}_{\mathrm{1,2}}={{\rm{\Delta }}}_{\mathrm{1,2}}[1+{\eta }_{C}{\cos ({\rm{\Omega }}}_{C}t)]+g{Q}_{\mathrm{1,2}}$. Note, in particular, that *M*_1,2_ and thus *M* is intrinsically time-dependent via *F*_1,2_ and therefore *A*_1,2_ [see Eq. (3)].

As proposed by Mari *et al*.^19^, we can gauge the synchronization level of indirectly coupled mechanical oscillators through a figure of merit

9 $$S(t)={\langle {q}_{-}^{2}(t)+{p}_{-}^{2}(t)\rangle }^{-1}$$

with the synchronization errors ${q}_{-}(t)=[{q}_{1}(t)-{q}_{2}(t)]/\sqrt{2}$ and ${p}_{-}(t)=[{p}_{1}(t)-{p}_{2}(t)]/\sqrt{2}$. In general, *S*(*t*) is contributed by both classical errors ${{Q}}_{1}(t)-{{Q}}_{2}(t)$ and ${P}_{1}(t)-{P}_{2}(t)$ irrelevant to the noise terms and quantum errors $\langle \delta {q}_{1}(t)-\delta {q}_{2}(t)\rangle$ and $\langle \delta {p}_{1}(t)-\delta {p}_{2}(t)\rangle$ arising from the noise terms. Then it is appropriate to use

10 $${S}_{q}(t)={\langle \delta {q}_{-}^{2}(t)+\delta {p}_{-}^{2}(t)\rangle }^{-1}$$

as a measure of the pure quantum synchronization with the classical contributions excluded. This quantum figure of merit has the maximal value 1.0 corresponding to the complete synchronization as limited by the Heisenberg’s uncertainty principle.

The calculation of ${S}_{q}(t)$ involves a few quadratic terms $\delta {q}_{j}^{2}(t)$, $\delta {q}_{1}\delta {q}_{2}(t)$, $\delta {p}_{j}^{2}(t)$, and $\delta {p}_{1}\delta {p}_{2}(t)$ so that we have to introduce a 8 × 8 covariance matrix

11 $${V}_{ij}(t)=\frac{1}{2}\langle {u}_{i}(t){u}_{j}(t)+{u}_{j}(t){u}_{i}(t)\rangle $$

and attain its dynamic equation ^8,29,38,39^

12 $$\dot{V}=MV+V{M}^{T}+N$$

directly from Eq. (5). In the above $N=diag[\mathrm{0,}\,{\gamma }_{m}\mathrm{(2}{n}_{b}+\mathrm{1),}\,\kappa ,\kappa ,\,\mathrm{0,}\,{\gamma }_{m}\mathrm{(2}{n}_{b}+\mathrm{1),}\,\kappa ,\kappa ]$ is a diagonalized 8 × 8 coefficient matrix answering for the correlation relation of noise operators and satisfying ${N}_{ij}\delta (t-{t}^{\text{'}})$ $=\langle {n}_{i}(t){n}_{j}({t}^{\text{'}})+{n}_{j}({t}^{\text{'}}){n}_{i}(t)\rangle /2$. Hence ${S}_{q}(t)$ can be expressed in a more concise form

13 $$\begin{array}{c}{S}_{q}(t)=\frac{1}{2}\{[{V}_{11}(t)+{V}_{55}(t)-{V}_{15}(t)-{V}_{51}(t)]\\ \,\,\,\,\,\,\,\,\,\,\,\,\,\,+{[{V}_{22}(t)+{V}_{66}(t)-{V}_{26}(t)-{V}_{62}(t)]\}}^{-1}\end{array}$$

Solving Eqs (3), (5) and (12) together under a given initial condition, it is then easy to examine the quantum synchronization of indirectly coupled mechanical oscillators. Note, however, that a good quantum synchronization is meaningful only when the optomechanical system is asymptotic stable, *i*.*e*., when all eigenvalues of the coefficient matrix *M* have negative real parts after a temporary evolutionary process according to the Routh-Hurwitz criterion^40^. In this regard, we would have a stable limit-cycle solution, representing a periodic oscillation, for ${{Q}}_{j}(t)$ and ${P}_{j}(t)$.

Finally, we introduce a widely used measure known as the Pearson factor for the classical synchronization^41–44^.

14 $${C}_{{Q}_{1},{Q}_{2}}(t,{\rm{\Delta }}t)=\frac{\overline{\delta {Q}_{1}\delta {Q}_{2}}}{\sqrt{\overline{\delta {Q}_{1}^{2}}\,\overline{\delta {Q}_{2}^{2}}}}$$

with $\delta {Q}_{i}={Q}_{i}-{\overline{Q}}_{i}$ and ${\overline{Q}}_{i}=\frac{1}{{\rm{\Delta }}t}{\int }_{t}^{t+{\rm{\Delta }}t}{Q}_{i}(t^{\prime} )dt^{\prime} (i=1,\mathrm{2)}$. The Pearson factor is bounded from 1.0 to −1.0 corresponding to the complete synchronization and the complete anti-synchronization, respectively. In fact, ${C}_{{{Q}}_{1},{{Q}}_{2}}$ and *S*_*q*_ are regarded here as the first-order and second-order synchronization criteria, respectively, for the two indirectly coupled mechanical oscillators.

## Results and Discussion

In this section, we examine via numerical calculations how to enhance the quantum synchronization in the presence of a good classical synchronization by periodically modulating the cavity modes or the driving fields. In what follows, we will use ${\overline{C}}_{{Q}_{1},{Q}_{2}}$ and ${\overline{S}}_{q}$ to represent the mean values of classical and quantum synchronizations after the system has evolved into the stable state^19^. We will also set ${{\rm{\Delta }}}_{j}={\omega }_{mj}$ to attain self-sustained mechanical oscillations (a prerequisite of synchronization) with blue-detuned driving fields^25,26,45^.

**Figure 2:**
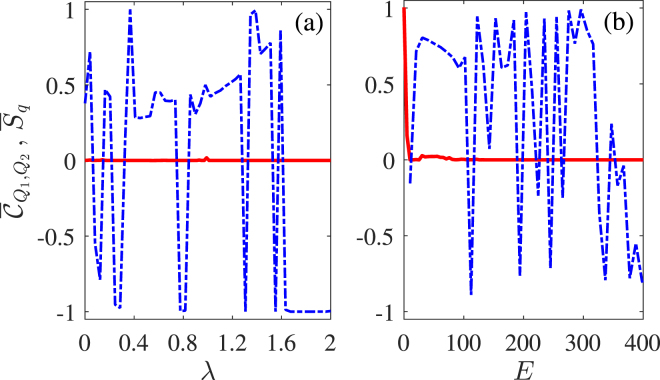
Mean values of quantum ${\overline{S}}_{q}$ (red-solid) and classical ${\bar{C}}_{{Q}_{1},{Q}_{2}}$ (blue-dashed) synchronization measures versus coupling constant *λ* (a); driving amplitude *E* (b) in the absence of external modulations. Relevant parameters are ${{\rm{\Delta }}}_{1}={\omega }_{m1}=1.0$, ${{\rm{\Delta }}}_{2}={\omega }_{m2}=1.005$, *g* = 0.005, ${\gamma }_{m}=0.005$, *k* = 0.15, *λ* = 0.05, and *E* = 100.

We start by considering the simple case without periodic modulations and illustrating relevant results in Fig. 2. It is clear that quantum synchronization is negligible in the absence of periodic modulations though it is possible to have rather good classical synchronization when the two cavity modes are coupled by an optical fiber and driven by two optical fields of identical amplitudes. To be more specific, ${\overline{C}}_{{Q}_{1},{Q}_{2}}$ may approach 1.0 when the coupling constant *λ* and the driving amplitude *E* are suitably chosen while ${\overline{S}}_{q}$ always tends to vanishing as long as *E* is not too small. When *E* is small enough, however, ${\overline{S}}_{q}$ may approach 1.0 while ${\overline{C}}_{{Q}_{1},{Q}_{2}}$ decreases greatly, indicating that the classical phase-space trajectory is not a limit cycle. So we choose *E* = 100 in the following calculations to guarantee limit-cycle solutions for our optomechanical system. In the regime of limit-cycle solutions, we then examine whether periodic modulations on cavity modes and driving fields^27,28,30^ can be exploited to enhance quantum synchronization of mechanical oscillators.

### Modulation on cavity modes

**Figure 3:**
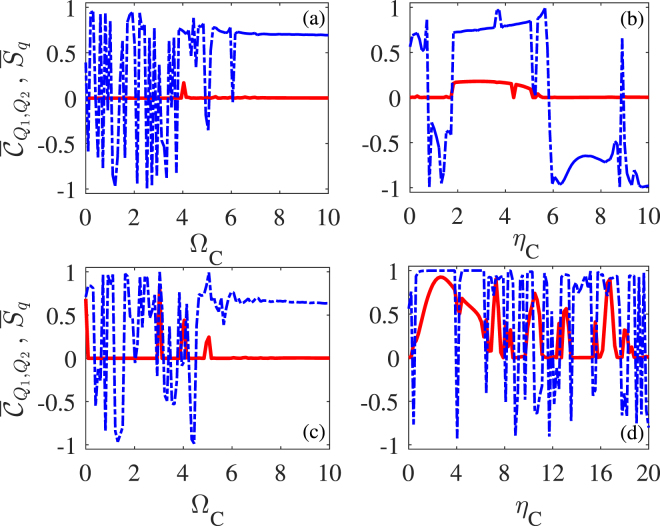
Mean values of quantum ${\overline{S}}_{q}$ (red-solid) and classical ${\bar{C}}_{{Q}_{1},{Q}_{2}}$ (blue-dashed) synchronization measures versus modulation frequency Ω_*C*_ with ${\eta }_{C}=2.0$ (**a**,**c**); modulation amplitude *η*_*C*_ with Ω_*C*_ = 4.0 (**b**) and Ω_*C*_ = 3.0 (**d**) which have been checked to be the optimal choice. A periodic modulation is applied upon only one cavity mode in panels (**a**,**b**) while upon both cavity modes in panels (**c**,**d**). Other parameters are the same as in Fig. 2.

We first consider the periodic modulation on cavity lengths and thus mode frequencies with, *e*.*g*., piezoelectric transducers attached to outside mirrors^30^. That is, the driving fields have a constant amplitude (${\eta }_{D}=\mathrm{0,}\,{{\rm{\Omega }}}_{D}=0$) while the cavity detunings vary periodically in time. We plot in Fig. 3 mean values ${\bar{C}}_{{Q}_{1},{Q}_{2}}$ and ${\overline{S}}_{q}$ for classical and quantum synchronizations as a function of ${\eta }_{C}$ or Ω_*C*_ for a single cavity-mode modulation (a, b) and a double cavity-mode modulation (c, d), respectively. Figure 3(a) and (b) show that the quantum synchronization can be slightly enhanced for appropriate values of ${\eta }_{C}$ or Ω_*C*_ in the presence of somewhat modified classical synchronization. Figure 3(c) and (d) show that quite good synchronization behaviors exist in both quantum and classical regimes for appropriate values of ${\eta }_{C}$ or Ω_*C*_. It is thus clear that double cavity-mode modulation has a considerable improvement in enhancing quantum synchronization as compared to single cavity-mode modulation. In particular, the optimal values are ${\overline{C}}_{{Q}_{1},{Q}_{2}}\approx 1.0$ and ${\overline{S}}_{q}=0.84$ at ${{\rm{\Omega }}}_{C}=3$ with ${\eta }_{C}=2$ in Fig. 3(c); ${\overline{C}}_{{Q}_{1},{Q}_{2}}\approx 1.0$ and ${\overline{S}}_{q}=0.92$ at ${\eta }_{C}=2.6$ with Ω_*C*_ = 3 in Fig. 3(d). We also find from Fig. 3(c) that good quantum synchronization occurs when Ω_*C*_ is an integral multiple of ${\omega }_{m}$ because in this case it is easier to transfer energy from external modulations to mechanical oscillations. But the peak positions may change from ${{\rm{\Omega }}}_{C}/{\omega }_{m}=3,4,5$ to other integers depending, e.g., on the value of ${\eta }_{C}$ (not shown). In addition, the modulation effect may sudden fail, i.e., ${\overline{S}}_{q}$ and ${\overline{C}}_{{Q}_{1},{Q}_{2}}$ become invariant, when Ω_*C*_ exceeds a critical value. Finally we find from Fig. 3(d) that, when ${\eta }_{C}$ is large enough, ${\overline{S}}_{q}$ and ${\overline{C}}_{{Q}_{1},{Q}_{2}}$ exhibit unstable oscillations as a result of the additional optomechanical instability due to parametric amplification^27^.

**Figure 4:**
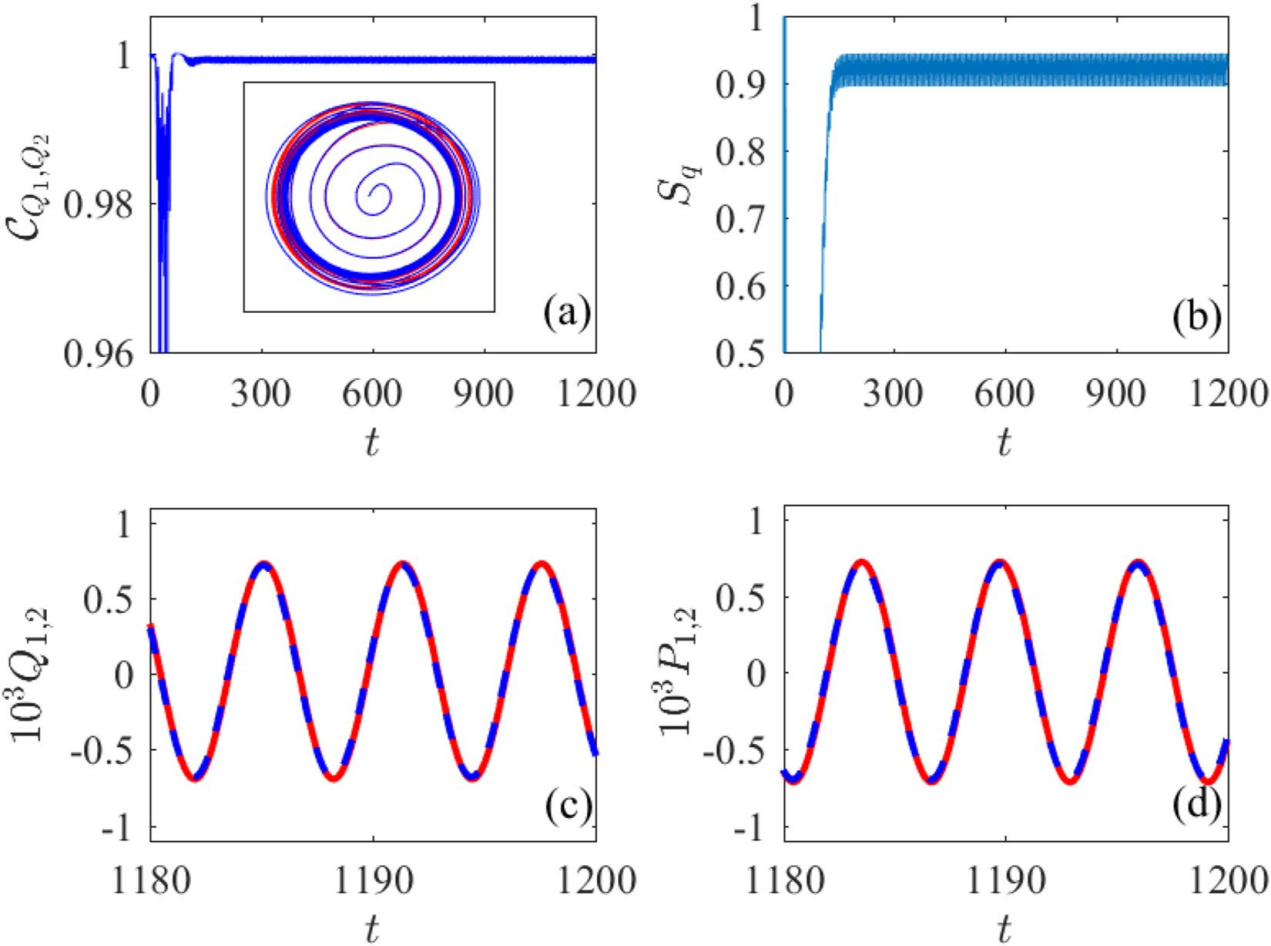
Time evolution of classical synchronization ${\bar{C}}_{{Q}_{1},{Q}_{2}}$ (**a**), quantum synchronization *S*_*q*_ (**b**), oscillator positions ${{Q}}_{\mathrm{1,2}}(t)$ (**c**), and oscillator momenta ${P}_{\mathrm{1,2}}(t)$ (**d**) in the case where both cavity modes are modulated with the optimal parameters Ω_*C*_ = 3.0 and ${\eta }_{C}=2.6$ as found in Fig. 3. Other parameters are the same as in Fig. 2.

To have a deeper insight into the synchronization behaviors, we further examine in Fig. 4 time evolutions of relevant mechanical variables and synchronization measures in the case of an optimal double cavity-mode modulation with Ω_*C*_ = 3 and ${\eta }_{C}=2.6$. Figure 4(a) and (b) show that both ${C}_{{Q}_{1},{Q}_{2}}$ and *S*_*q*_ reach a stable state of slight oscillation after a (different) transient evolution. As a further evidence, classical positions *Q*_1_ and *Q*_2_ are found to oscillate exactly in phase when entering the stable state as shown in Fig. 4(c). The same conclusion holds for classical momenta *P*_1_ and *P*_2_ as shown in Fig. 4(d). Therefore, by periodically modulating cavity detunings in a suitable way, it is viable to produce a rather ideal level of both quantum and classical synchronizations between two mechanical oscillators with different frequencies. Corresponding limit-cycle trajectories in the ${P}_{1}\rightleftharpoons {Q}_{1}$ (red) and ${P}_{2}\rightleftharpoons {Q}_{2}$ (blue) spaces are illustrated in the inset of Fig. 4(a).

### Modulation on driving fields

**Figure 5:**
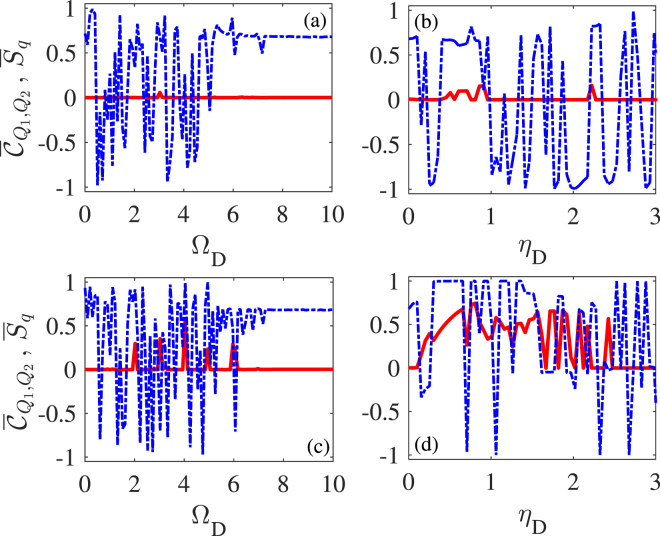
Mean values of quantum ${\overline{S}}_{q}$ (red-solid) and classical ${\overline{C}}_{{Q}_{1},{Q}_{2}}$ (blue-dashed) synchronization measures versus modulation frequency Ω_*D*_ with ${\eta }_{D}=0.5$ (**a**,**c**); modulation amplitude *η*_*D*_ with Ω_*C*_ = 3.0 (**b**) and Ω_*C*_ = 4.0 (**d**) which have been checked to be the optimal choice. A periodic modulation is applied upon only one driving field in panels (**a**,**b**) while upon both driving fields in panels (**c**,**d**). Other parameters are the same as in Fig. 2.

We then consider the periodic modulation on amplitudes of the driving fields, *e*.*g*., via acousto-optical modulators. That is, the cavity modes have a constant detuning (${\eta }_{C}=\mathrm{0,}\,{{\rm{\Omega }}}_{C}=0$) while the driving amplitudes vary periodically in time. We plot in Fig. 5 mean values ${\overline{C}}_{{Q}_{1},{Q}_{2}}$ and ${\overline{S}}_{q}$ for classical and quantum synchronizations as a function of ${\eta }_{D}$ or Ω_*D*_ for a single driving-amplitude modulation (a, b) and a double driving-amplitude modulation (c,d), respectively. Once again we find that (i) quantum synchronization can be slightly enhanced with somewhat modified classical synchronization in the case of single driving-amplitude modulation; (ii) both quantum and classical synchronizations are quite satisfactory in the case of double driving-amplitude modulation. In particular, the optimal values are ${\bar{C}}_{{Q}_{1},{Q}_{2}}\approx 1.0$ and ${\overline{S}}_{q}=0.57$ in Fig. 5(c); ${\bar{C}}_{{Q}_{1},{Q}_{2}}\approx 1.0$ and ${\bar{S}}_{q}=0.74$ in Fig. 5(d). By comparing Fig. 5(c,d) with Fig. 3(c,d), it is clear that double cavity-mode modulation is more favorable than double driving-field modulation for achieving an ideal level of quantum and classical synchronizations. One common feature of double driving-field and cavity-mode modulations is that optimal quantum synchronization occurs when the modulation frequency is an integral multiple of the oscillator frequency before a critical value.

**Figure 6:**
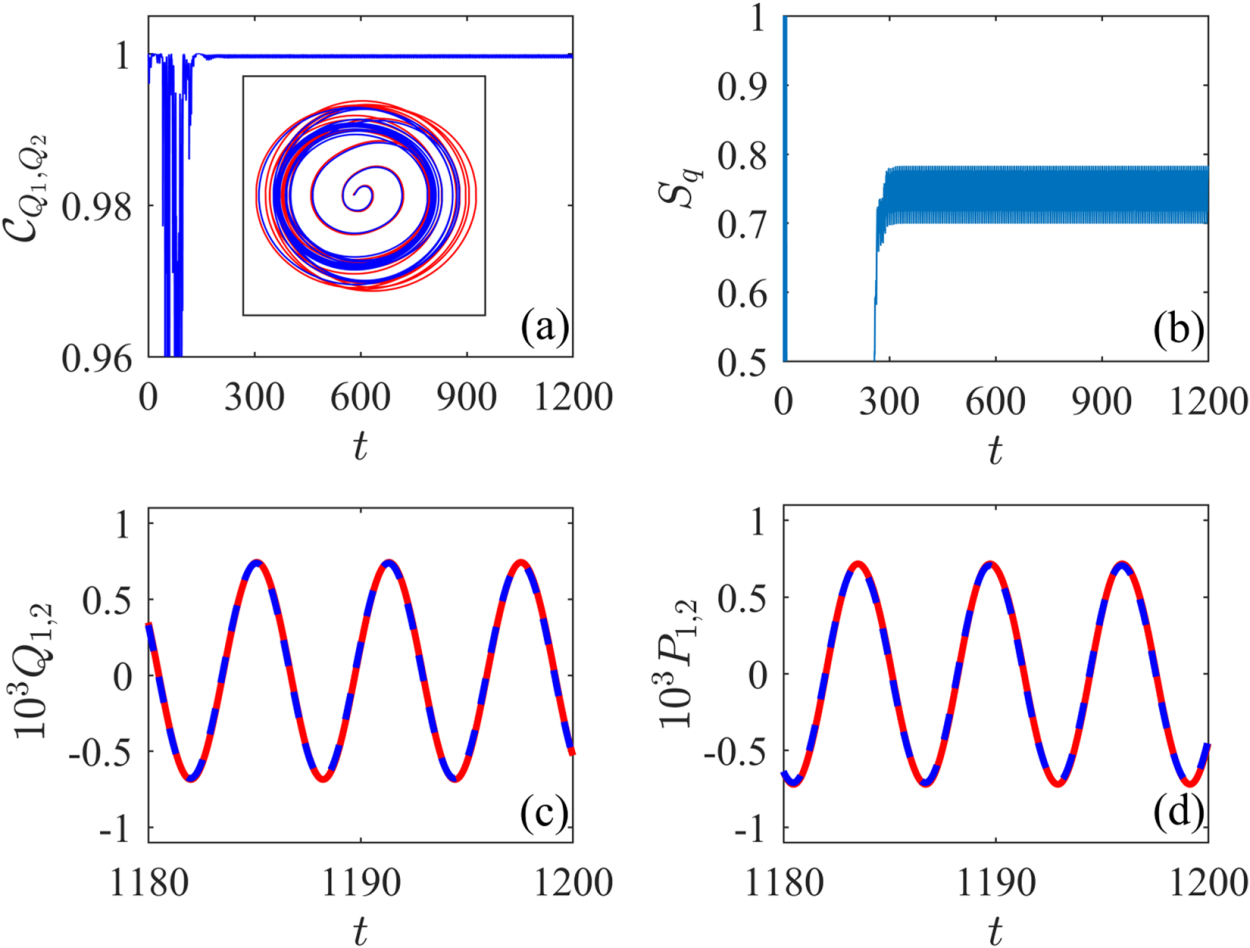
Time evolution of classical synchronization ${C}_{{Q}_{1},{Q}_{2}}$ (**a**), quantum synchronization *S*_*q*_ (**b**), oscillator positions ${Q}_{\mathrm{1,2}}(t)$ (**c**), and oscillator momenta ${P}_{\mathrm{1,2}}(t)$ (**d**) in the case where both driving fields are modulated with the optimal parameters Ω_*C*_ = 4.0 and ${\eta }_{D}=0.8$ as found in Fig. 5. Other parameters are the same as in Fig. 2.

We further show in Fig. 6 that the time evolution of relevant mechanical variables and synchronization measures in the case of an optimal double driving-amplitude modulation with Ω_*D*_ = 4 and ${\eta }_{D}=0.8$. From Fig. 6(a) and (b) we can see that both ${\bar{C}}_{{Q}_{1},{Q}_{2}}$ and *S*_*q*_ reach a stable state of slight oscillation after a (different) transient evolution, longer than that in Fig. 4(a) and (b). As a further evidence, classical positions *Q*_1_ and *Q*_2_ and classical momenta ${P}_{1}(t)$ and ${P}_{2}(t)$ are found to oscillate exactly in phase when entering the stable state as shown in Fig. 6(c) and (d). Therefore, by periodically modulating driving amplitudes in a suitable way, it is also viable to produce very good quantum and classical synchronizations between two mechanical oscillators with different frequencies. Two corresponding limit-cycle trajectories are illustrated in the inset of Fig. 6(a).

### Comparison of two modulations

Now we examine the robustness of quantum synchronization in both cases of cavity-mode and driving-field modulations against the bath’s mean temperature *T* and the oscillators’ frequency difference Δ_*m*_. This is based on the consideration that a slight increase of *T* and ${{\rm{\Delta }}}_{m}$ may result in a large decrease of ${\overline{S}}_{q}$ so that it is meaningful to check how ${\overline{S}}_{q}$ decays until negligible.

**Figure 7:**
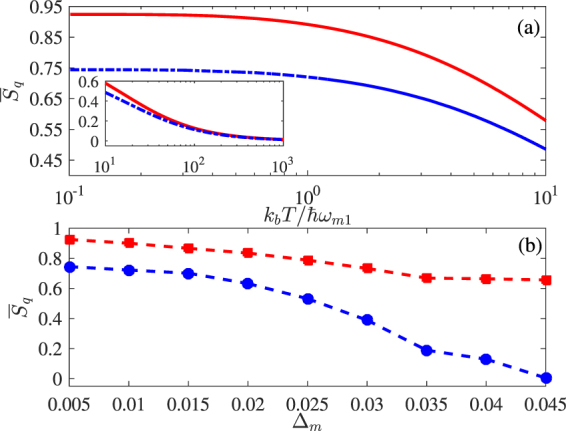
Mean values of quantum synchronization *S*_*q*_ versus the bath’s mean temperature *T* (**a**); the oscillators’ frequency difference Δ_*m*_ (**b**) for an optimal cavity-mode modulation (red-solid) or an optimal driving-field modulation (blue-dashed). Relevant parameters except Ω_*C*,D_ and *η*_*C*,*D*_ are the same as in Fig. 2.

We plot ${\overline{S}}_{q}$ versus mean temperature *T* in Fig. 7(a) and frequency difference Δ_*m*_ in Fig. 7(b) for the optimal modulations on cavity detunings (red-solid) or driving amplitudes (blue-dashed). That is, each point represents the maximal value of ${\overline{S}}_{q}$, for a given value of *T* or Δ_*m*_, obtained by choosing the optimal values of ${\eta }_{C}$ and Ω_*C*_ or ${\eta }_{D}$ and Ω_*D*_. Figure 7(a) shows that the quantum synchronization is quite robust (*i*.*e*., does not change too much) against the temperature before $T \sim \hslash {\omega }_{m1}/{k}_{b}$. However, it decays quickly after this point and tends to be vanishing when the temperature is around $T=1000\hslash {\omega }_{m1}/{k}_{b}$. It is also clear that the optimal modulation on cavity modes always results in a better quantum synchronization than that on driving fields. Figure 7(b) shows that the quantum synchronization ${\overline{S}}_{q}$ is quite robust against the frequency difference Δ_*m*_ for an optimal cavity-mode modulation because ${\overline{S}}_{q}$ doesn’t decrease too much even if Δ_*m*_ increases from 0.005 to 0.045. However, the quantum synchronization ${\overline{S}}_{q}$ decays in a much quicker way for an optimal driving-field modulation and already exhibits a vanishing value around Δ_*m*_ ~ 0.045. It is also worth noting that the optimal level of quantum synchronization observed here (~0.92 or ~0.74) is much higher than that for two directly coupled oscillators (~0.3)^19^ for the same frequency difference Δ_*m*_ = 0.005.

## Conclusions

In summary, we have considered a double-cavity optomechanical system containing two independent mechanical oscillators for enhancing both quantum and classical synchronizations with two kinds of temporal periodic modulation. Our numerical results show that appropriate modulations on cavity detunings or driving amplitudes can result in greatly enhanced quantum and classical synchronizations. To be more specific, the quantum synchronization ${\overline{S}}_{q}$ can be up to ~0.92 (~0.74) in the case of cavity-detuning (driving-amplitude) modulation accompanied with a roughly perfect classical synchronization ${\bar{C}}_{{Q}_{1},{Q}_{2}}\approx 1$ when the oscillators’ frequency difference is Δ_*m*_ = 0.005 and the bath’s mean temperature is *T* = 0. An examination of the robustness of ${\overline{S}}_{q}$ against Δ_*m*_ and *T* shows that the cavity-mode modulation is always more appealing in achieving a preferable quantum synchronization behavior than the driving-field modulation. We expect that our results may be extended to more complicated multi-cavity optomechanical systems, in which an array of highly synchronized mechanical oscillators can serve as a useful resource of, *e*.*g*., quantum communication and quantum control.
